# Innovative Therapeutic Approaches in Primary Cutaneous B Cell Lymphoma

**DOI:** 10.3389/fonc.2020.01163

**Published:** 2020-08-07

**Authors:** Claudia C. V. Lang, Egle Ramelyte, Reinhard Dummer

**Affiliations:** ^1^Department of Dermatology, University Hospital Zurich, Zurich, Switzerland; ^2^Medical Faculty, University of Zurich, Zurich, Switzerland

**Keywords:** CBCL, radiotherapy, T-VEC, dacetuzumab, bruton thyrosine kinase, intralesional interferon alpha

## Abstract

**Background:** Primary cutaneous B-cell lymphomas (pCBCL) include an infrequent group of non-Hodgkin lymphomas that are limited to skin sites at the time of diagnosis. They comprise roughly 20–25% of all cutaneous lymphomas and are subdivided into primary cutaneous marginal zone lymphoma (PCMZL), primary cutaneous follicle center lymphoma (PCFCL), and primary cutaneous diffuse large cell B cell lymphoma, leg type (PCDLCBCL, LT). The first two show a rather indolent course while PCDLCBCL, LT carries a worse prognosis. Intravascular large cell B-cell lymphoma is the most infrequent subtype, and its therapy is not covered in this review.

**Topical Therapy:** For solitary, single-site PCMZL and PCFCL, several topical treatment options exist. They include, but are not limited to, excision, radiotherapy, and intralesional therapies, discussed in this review. However, in selected cases, even “watchful waiting” is reasonable.

**Systemic Therapy:** Indolent types of pCBCL rarely require systemic treatment. However, in extended cases and more importantly DLCBCL, LT, systemic treatment is the first choice. Monoclonal anti-CD20-antibody rituximab is often used as monotherapy in PCMZL and PCFCL or combined with chemotherapy in PCDLBCL, LT. Newer options are monoclonal anti-CD40 antibody dacetuzumab, anti-PD-1 and anti-PD-L1 checkpoint inhibitors, and Bruton tyrosine kinase inhibitors.

**Conclusion:** Indolent pCBCL are treated with a risk-adapted strategy using intralesional steroids, RT, and interferon-α as first-line treatments. Relapsing cases may profit from rituximab. In aggressive PCDLCBCL, LT, rituximab with polychemotherapy is recommended. Innovative therapies include intralesional oncolytic virotherapy, systemic monoclonal antibodies, and small molecules.

## Introduction

Primary cutaneous lymphomas are a group of extranodal non-Hodgkin lymphomas, which present in the skin without signs of systemic involvement at the time of diagnosis (1). The incidence in Western countries is 1 in 100,000, 20–25% of which are primary cutaneous B-cell lymphomas (pCBCL) (2). Most common pCBCL are primary cutaneous marginal zone lymphoma (PCMZL), primary cutaneous follicle center lymphoma (PCFCL), and primary cutaneous diffuse large B-cell lymphoma, leg type (PCDLBCL, LT). Even though all three entities arise from B-cells, they show different clinical courses and carry different prognoses. PCMZL and PCFCL are considered low-grade lymphomas. Despite the high rate of cutaneous relapses, they respond well to therapies and usually have an excellent prognosis. PCDLBCL, LT is a high-grade lymphoma, which carries a worse prognosis and usually requires aggressive treatment approaches.

For localized low-grade lymphomas, skin-directed therapies with surgery, intralesional corticosteroids or interferon-α, and radiotherapy are vivid treatment options (3, 4). Despite high initial response rates, relapses are common. In generalized, relapsing, and advanced cases, systemic chemotherapy or immunotherapy is required. In aggressive cases, such as PCDLBCL, LT, a combination chemotherapy with monoclonal antibodies, i.e., R-CHOP (rituximab, cyclophosphamide, doxorubicin, vincristine, prednisone) is the most common implemented therapy. However, over 50% of patients fail to respond to therapy or, more commonly, develop a relapse after initial response (5, 6). Moreover, aggressive chemotherapy can seldom be administered in elderly frail patients. These limitations along with the observed similar response pattern to skin-directed and systemic therapies support the need of new therapeutic approaches for patients with widespread, refractory, and relapsing diseases.

## Topical Therapies

### Excision

Surgical excision is among the recommended first-line therapies for solitary pCBCL lesions (4). Senff et al. reported on 75 PCMZL patients treated with excisions. In this cohort, all but one patient achieved complete response; however, 43% of patients developed a relapse. As the site of relapse (local recurrences vs. relapse on other sites) was not reported, the actual cure rate remains unclear; also a clear definition of safety margins in the literature is missing (6).

### Radiotherapy

Along with the excision, radiotherapy (RT) is recommended as a first-line therapy for solitary lesions. It is also a good treatment option for systemic therapy-resistant pCBCL lesions. Different types of RT can be implemented, including electrons and low-energy X-rays. If treated with electrons, the required energy recommendation ranges between 6 and 9 MeV for depth coverage. Low-energy X-rays (~100 kV) may be used alternatively. The recommended total dose range varies between 20 and 36 Gy for PCMZL and PCFCL (NCCN recommends 24 to 30 Gy; EORTC/ISCL recommend 20 to 36 Gy for PCMZL and >30 Gy for PCFCL).

Recommendations for safety margins with RT also vary in the literature and ranges between 0.5 and 5.0 cm. The EORTC/ISCL and ILROG recommend a margin of 1.0 to 1.5 cm for PCMZL and PCFCL (6).

Due to the high relapse rate, even solitary and localized PCDLBCL, LT lesions should be treated with first-line R-CHOP followed by localized RT with safety margins of 1–2 cm. For electrons, an energy of 6–9 MeV can be used, and a dose of 36–40 Gy is recommended. In cases without systemic pretreatment, a full dose of 40 Gy should be applied (7).

In a palliative regimen for pCBCL, low-dose RT can be applied with a total dose of 4 Gy given in two fractions within 2–4 days. Neelis et al. showed that, with this regimen, complete response at 4–6 weeks could be achieved in 75% of the 18 treated pCBCL patients with 44 different lesions in total (8).

### Antibiotics

Even though the literature around the role of *B. burgdorferi* in PCMZL is not conclusive, PCR examination is still positioned in routine workups, and the association should be further investigated (4). In *B. burgdorferi* infection-associated PCMZL, antibiotic therapy should be attempted before more aggressive therapies are used. However, the literature regarding indication, efficacy, and antibiotic treatment course is not univocal. Senff et al. report in their review about 14 patients, of which 43% achieved complete remission after various antibiotic regimens (cephalosporins, e.g. cefuroxime 500 mg bid, and tetracyclines, e.g. doxycycline 100 mg bid, both usually given over 3 weeks). In a small cohort of eight patients, they specify intravenous treatment with cephalosporins seems to be superior to oral treatment with high-dose tetracyclines (6).

### Intralesional Regimens

For solitary PCMZL and PFBCL lesions, intralesional regimens are simple, cost-effective, and efficient treatment options. Intralesional steroids, e.g., triamcinolone diluted with lidocaine, lead to lesion size reduction within 2–3 applications with a 3- to 4-weeks interval. There are a few side effects except for the risk of skin atrophy (9). Only limited data on intralesional treatment with interferon-α (IFN-α) is available. One study reported successful use of intralesional IFN-α in eight PCMZL patients. The dosing of 3 million IU three times per week led to complete remission after a median of 8 weeks (10). The intralesional administration of rituximab was only administered in nine patients with 5–30 mg once or three times a week, of which eight (89%) reached complete remission with a 62% relapse rate (6).

### Modified Viruses

Genetically modified viruses are an upcoming intralesional treatment option. For pCBCL, there are two different virus types available: non-replicating viruses used as vectors (adenovirus interferon-γ) and replicating viruses (Talimogene laherparepvec). Dreno et al. performed a phase II open-label multicenter study with repeated intralesional administration of adenovirus interferon-γ (TG1042). TG1042 is an adenovirus five expressing the cDNA of the human IFN-γ gene. The virus was chosen due to a short half-life and significant side effects upon systemic treatment with cytokine IFN-γ. Thirteen patients were enrolled, and 11 (85%) showed an objective response with a median progression-free survival of 23.5 months. The intralesional administration of TG1042 showed most commonly minor to moderate flu-like symptoms (11).

An ongoing phase I clinical trial is exploring intralesional talimogene laherparepvec (T-VEC) in non-melanoma skin cancer, including cutaneous lymphomas (NCT03458117). T-VEC is a genetically engineered Herpes simplex 1 virus, which is modified to replicate in tumor cells and stimulate adaptive immunity. In metastatic melanoma, it caused responses not only in injected, but also in non-injected metastases (12, 13). The same treatment regimen is used in the clinical trial with initial injections of 10^6^ PFU/ml followed by 10^8^ PFU/ml every 2 weeks.

### Topical Imiquimod

Topical imiquimod can be used as an immunomodulator via activation of the transcription factor NF-κB via the *Toll-like receptor 7* (TLR7) and *Toll-like receptor 8* (TLR8) signaling pathways. This activation leads to production of pro-inflammatory mediators, such as IFN-α, IFN-γ, interleukin 12, and tumor necrosis factor-α, which activate antigen-presenting cells and induce T-helper 1 (Th1) antitumoral cellular immune response (14). There are several case reports and a few small studies available showing topical imiquimod as an option in indolent pCBCL; however, the results are more promising for primary cutaneous T cell lymphomas (pCTCL) than pCBCL. The treatment frequency ranged from once daily to once weekly applications and was generally well-tolerated; time to response was reached after 2 weeks on average. Severe reactions mostly developed in patients on an intensive regimen and could be avoided with less frequent applications (15).

### Photodynamic Therapy

Photodynamic therapy (PDT) is the combination of a photosensitizer, e.g., 5-aminolevulinic acid (5-ALA, or its methyl ester), light, and oxygen. The photosensitizer is applied to the treatment area, accumulates in abnormal skin tissue, and converts it into protoporphyrin IX. In the presence of light (visible to infrared spectrum), it leads to a photochemical and phototoxic reaction using free oxygen radicals, which leads to cell death. The common use for PDT is mainly for treatment of non-melanoma skin cancer and field cancerization (16). There are only a few case reports and studies for the use in primary cutaneous lymphoma, mainly for pCTCL (15). Mori et al. treated three patients with single early pCBCL lesions using 5-ALA and red light within a standard dose setting for basal cell carcinoma. All three patients reached complete remission after a single session with a follow- up period between 8 and 17 months (17).

## Systemic Therapies

### Monoclonal Antibodies

#### Rituximab and Versions of It

CD20 is a B cell differentiation antigen, expressed as a surface molecule on all B lineage cells. Rituximab is the first chimeric monoclonal antibody (mAb) that showed positive results in systemic B cell malignancies and also cutaneous B-cell lymphomas (18). It binds to CD20 on malignant and benign B-cells and elicits its effect through cell apoptosis, antibody-dependent cellular cytotoxicity (ADCC), and complement-mediated cytotoxicity (19). Used as monotherapy in a small subset of pCBCL patients, rituximab resulted in an overall response rate (ORR) of 89%. However, within a median 52-months of follow up, 81% patients developed a relapse at a median time to relapse of 25 months (18). However, no larger clinical trials in pCBCL have been conducted so far (20).

In advanced pCBCL and nearly all DLCBCL, LT cases, a combination of immunochemotherapy with R-CHOP is the first-line treatment. An observed complete response to CHOP-like chemotherapy in patients with DLCBCL, LT reaches 81% with a subsequent relapse in 56% cases. These results are reported from 32 patients treated in eight separate clinical trials (6) as, even though commonly implemented, only limited cases of R-CHOP in pCBCL are reported.

In refractory cases, rituximab can also be applied in combination with other systemic therapies. A patient with DLCBCL, LT, who relapsed after radiotherapy and R-CGOP (rituximab, cyclophosphamide, gemcitabine, vincristine, prednisone), was treated with a combination of rituximab, lenalidomide antiPD-1 antibody pembrolizumab (21). The patient developed a complete response, which was still ongoing at 7 months after the initiation of therapy. Given this complex immune-modulating treatment, the patient developed a transient neutropenia and pneumonia, which responded to antibiotic therapy.

A new generation of higher affinity, lower immunogenicity, and increased efficacy anti-CD20 antibodies are being investigated. Among those are also antiCD20 mAbs with radioactive molecules. This approach with antiCD20-Mab IgG-radioconjugates Y-ibrutumab (Ibritumomab tiuxetan) and I-tositumomab (Iodine I 131 Tositumomab) is an already FDA approved treatment for nodal B-cell lymphoma (22, 23) and might be applied in advanced pCBCL cases.

#### Other Monoclonal Antibodies

CD40 is a member of the TNF-receptor superfamily and is expressed on healthy B-cells as well as some B-cell neoplasms, such as chronic lymphocytic leukemia, non-Hodgkin lymphoma, and multiple myeloma. It functions as a co-stimulatory molecule upon the interaction with CD40-ligand (CD40L) and CD154 on T-cells (24, 25). It plays a critical role in stimulating antigen presentation, priming of helper- and cytotoxic T-cells and various inflammatory reactions. In cancer, it is involved in the regulation of cancer-cell survival and the presentation of tumor-associated antigens (24).

Dacetuzumab (SNG-40) is a humanized IgG1 anti-CD40-Mab. *In vitro*, dacetuzumab acts like a partial agonist and does not prevent CD40/CD40L interaction. It also has little effect on normal B-cells, but kills malignant B-cells through ADCC and antibody-dependent phagocytosis (26). In a phase II clinical trial with dacetuzumab monotherapy in relapsed systemic DLCBCL patients, it demonstrated a modest overall response rate of 9% and had relatively a high rate of grade 3–4 lymphopenia (41%). However, it achieved disease control in 37% of patients, which suggests this medication may be adapted in different dosing or in combination with other therapeutics and may have a role in treatment of B-cell malignancies, including pCBCL (27).

The presence of myeloid-derived suppressor cells (MDSC) in the tumor microenvironment correlates to worse prognosis in solid tumors. In DLCBCL, LT, CD33-positive MDSC are present. This could explain the poor prognosis but also provides an opportunity for blockage with humanized anti-CD33 Mab, which has already shown positive results in acute myeloid leukemia (28). No clinical trials that include pCBCL patients are ongoing yet.

### Bispecific T-Cell Engaging (BiTE) Antibodies

A bispecific T-cell engaging (BiTE) antibody is a protein, which can bind two different antigens simultaneously. In B-cell malignancies, an antiCD19/CD3 mAb—blinatumomab—is being explored. CD19 is a differentiation antigen, expressed on the B-cells during all differentiation phases except for plasma cells. Blinatumomab is a 55-kDa fusion protein that simultaneously links CD3 and CD19. A transient cytolytic synapsis between a cytotoxic T-cell and a CD19-positive cell is formed, which results in the lysis of the CD19 cell (29, 30). Therapy with blinatumomab is currently focused on B-precursor acute lymphoblastic leukemia, but promising results were also obtained in relapsed or refractory DLCBCL, LT (31). However, it might not be suitable for advanced PCMZL cases as high numbers of plasma cells might be observed in some PCMZL cases. However, another BiTe, XmAb13676, which binds CD20/CD3, is currently being investigated in a phase I clinical trial in patients with CD20-expressing hematologic malignancies (NCT02924402).

### Checkpoint Inhibitors

Immunotherapies are playing an increasing role in modern cancer therapy. Immune response and T-cell activation is a highly regulated process. Checkpoints, such as programmed death 1 (PD-1) are expressed on the surface of T-cells and facilitate sending inhibitory feedback signals to control the cytotoxic T-cell activity and autoimmunity. In solid tumors, PD-1 and PD-ligand 1 (PD-L1) inhibitors show impressive responses (32) and significantly prolong overall survival compared to previously used therapies. Checkpoint inhibitors are also used in lymphocytic malignancies. In Hodgkin's lymphoma, increased PD-L1 expression on the malignant Hodgkin Reed Sternberg cells results from genetic amplification at chromosome 9p24.1 (33). In a group of treatment naïve and pretreated patients, anti-PD-1 monotherapy resulted in ORR of 69% with CR in 16% of patients (34).

In pCBCL, gene alterations, which result in PD-L1/PD-L2 upregulation were identified in DLCBCL, LT, but not in low-grade lymphomas (35). However, the biological function of PD-1/PD-L1 expression is less clear in lymphocytic malignancies. In pCTCL, where the meaning of PD-1/PD-L1 expression is also less clear, antiPD1 antibody pembrolizumab was tested in a clinical trial and resulted in 38% objective response rate in heavily pretreated cutaneous pCTCL patients (36).

Interestingly, in contrast to solid tumors, anti-PD-1 efficacy in pCTCL did not correlate with the expression of PD-L1, total mutational burden, or an interferon-gamma gene expression signature (36), which suggest that indolent pCBCLs may also respond without having the upregulated PD-1/PD-L1.

### Small Molecules

#### BTK Inhibitors

Bruton Tyrosine Kinase (BTK) is a cytoplasmic, non-receptor tyrosine kinase, predominantly expressed in B-lymphocytes, myeloid cells, and platelets. The BTK pathway is involved in B-cell receptor (BCR) signaling and plays a role in cell proliferation and survival. It is activated in multiple malignancies, including chronic lymphocytic leukemia, acute lymphoblastic leukemia, mantle cell lymphoma, diffuse large B-cell lymphoma, and others (37–41).

Ibrutinib is a first-in-class irreversible BTK inhibitor, which binds to the kinase by a covalent bond and inhibits continuous signaling, thus inhibiting cell proliferation. It is approved for lymphocytic malignancies with activated BKT signaling, including CLL (42), mantle cell lymphoma (43), and marginal zone lymphoma (44). A clinical trial explored the efficacy of ibrutinib in relapsed and/or refractory DLCBCL patients. The ORR was 25% (20/80) with CR in eight patients and partial response (PR) in 12 patients (45). In DLCBCL, LT, an 80-year-old patient was reported to achieve a complete clearance of skin involvement after ibrutinib treatment (46). However, the patient developed nodal disease 2 months after treatment start.

As seen in other B-cell malignancies, acquired resistance to BTK inhibitors is often caused by mutant BTK^C481S^. In BTK^C481S^ the covalent bond between BTK and the inhibitor is disrupted, which results in reduced drug efficacy. XMU-MP-3 is a low-molecular-weight, non-covalent BTK inhibitor, which has been shown to bind and inhibit both BTK and BTK^C481S^ *in vitro* and *in vivo* (47). So far, no clinical trials have been initiated.

#### Lenalidomide

Lenalidomide is an immunomodulatory drug used in multiple myeloma. Lenalidomide enhances the activity of cytotoxic T cells and natural killer cells. The upregulation of tumor-suppressor genes results in inhibition of cell signaling engaging NK-kB and IFN-βsignaling, which elicits antiproliferative and antiangiogenic effects (40, 48). Lenalidomide was explored as monotherapy in DLCBCL, LT, where it achieved a 6-months overall response rate in 26.3% of patients. Four out of 19 patients have achieved CR, and eight (42%) have achieved disease control (49). As mentioned above, it shows promising results in combination with other treatments, such as immunotherapy.

#### Venetoclax

BCL-2 is a protein that inhibits apoptosis by preventing the release of cytochrome c from the mitochondrial intermembrane space. The *t*_(14, 18)__(q32.3;q21.3)_ chromosomal translocation, which is common in systemic follicle center lymphoma, results in constitutive expression of BCL-2 and increased resistance to apoptosis (50). BCL-2 inhibitor Venteloclax (ABT-199) binds to BCL-2 protein and causes events, which activate BAX and BAK and subsequently induce apoptosis (51, 52). Venetoclax shows remarkable clinical activity in chronic lymphocytic leukemia; however, despite high-level expression of BCL-2 in systemic DLBCL, responses in this malignancy are seldom (53). It is possible, that co-expression of other anti-apoptotic BCL-2 family proteins may limit activity, and addition of other agents must be needed in DLCBCL Venetoclax has not been explored in pCBCL, but similar approaches with the need of combination therapy may be required.

## Conclusion

Localized pCBCL (PCMZL, PCFCL) shows high 5-yeara disease-specific survival rates (99 vs. 95%, respectively); hence, an active “wait and see” approach is often applicable. Skin-directed treatment regimens are first-line options for PCMZL and PCFCL, but all patients should be monitored for disease relapse independent of the applied treatment.

When it comes to PCDLBCL, LT, a quick and effective treatment start is crucial. First-line treatment is a systemic R-CHOP regimen with adjuvant radiotherapy. If poly-chemotherapy is contraindicated, disease control can be achieved with rituximab monotherapy in combination with radiotherapy for special cases. Radiotherapy alone only plays a role in palliative care regimens.

Systemic treatments almost exclusively play a role in PCDLBCL, LT, and systemic (extracutaneous) lymphoma. In resistant/relapsing cases or in patients with extensive cutaneous disease, systemic therapy can be considered although clinical data is sparse. A combination of systemic therapy with topical regimens can be applied for skin manifestations, mostly localized RT.

For topical regimens, there is a new approach with the intralesional application of T-VEC, currently investigated in a phase I trial. Otherwise, there are known, reliable treatments with interferon-α or rituximab. Overall, more randomized trials are needed to investigate treatment responses.

The field of systemic treatment is getting more interesting with a growing number of monoclonal antibodies, checkpoint inhibitors (PD1), Bruton kinase inhibitors and other small molecules. Although most of these agents are not registered for pCBCL yet and are only available in off-label or compassionate use settings. The active use of those agents implies a careful choice of patients, which should be made in a multi-interdisciplinary setting of a certified tumor board.

It is challenging to interest pharma companies to invest in trials for these rare indications. Therefore, high-impact numbers are difficult to reach, and data on new and innovative treatments are limited.

## Author Contributions

All authors listed have made a substantial, direct and intellectual contribution to the work, and approved it for publication.

## Conflict of Interest

The authors declare that the research was conducted in the absence of any commercial or financial relationships that could be construed as a potential conflict of interest.
